# Common pathogenic bacteria-induced reprogramming of the host proteinogenic amino acids metabolism

**DOI:** 10.1007/s00726-023-03334-w

**Published:** 2023-10-09

**Authors:** Xiao-yue Li, Zi-xin Zeng, Zhi-xing Cheng, Yi-lin Wang, Liang-Jun Yuan, Zhi-yong Zhai, Wei Gong

**Affiliations:** 1 The First School of Clinical Medicine, Southern Medical University, Guangdong, 510515 China; 2 Shenzhen Hospital, Southern Medical University, Shenzhen Clinical Medical College, Southern Medical University, Guangdong, 518101 China

**Keywords:** Amino acids, Metabolic reprogramming, Bacterial infections, Nutrition

## Abstract

Apart from cancer, metabolic reprogramming is also prevalent in other diseases, such as bacterial infections. Bacterial infections can affect a variety of cells, tissues, organs, and bodies, leading to a series of clinical diseases. Common Pathogenic bacteria include *Helicobacter pylori*, *Salmonella enterica*, *Mycobacterium tuberculosis*, *Staphylococcus aureus,* and so on. Amino acids are important and essential nutrients in bacterial physiology and support not only their proliferation but also their evasion of host immune defenses. Many pathogenic bacteria or opportunistic pathogens infect the host and lead to significant changes in metabolites, especially the proteinogenic amino acids, to inhibit the host’s immune mechanism to achieve its immune evasion and pathogenicity. Here, we review the regulation of host metabolism, while host cells are infected by some common pathogenic bacteria, and discuss how amino acids of metabolic reprogramming affect bacterial infections, revealing the potential adjunctive application of amino acids alongside antibiotics.

## Introduction

Since the Warburg effect was first proposed in the 1920s (Liberti and Locasale 2016), metabolic reprogramming has been widely recognized as one of the hallmarks of cancer and even has a tendency to serve as the core one (Ward and Thompson 2012; Koppenol et al. 2011), whose main connotation is to promote rapid cell growth and proliferation by regulating energy metabolism. As metabolic research has become one of the forefront of academic research in recent years, metabolic reprogramming has also transformed from the proprietary concept of cancer research to the upstart in other fields (Hochrein et al. 2022), for example, the infectious diseases (Arshad et al. 2022).

Toxins and other metabolites, produced by pathogenic bacteria or opportunistic pathogens, can lead to infectious diseases which are considered as one of the leading causes for death worldwide annually (10.1038/s41564-018-0331-3), of which pathogenic microorganisms include bacteria, viruses, fungi, mycoplasma, etc. The acquisition of host nutrients by microorganisms is an essential step of infectious disease (Appelberg 2006; Rohmer et al. 2011). With the growth of inflammation and local hypoxia, pathogens are exposed to a complex and dynamic host nutritional microenvironment (Abu Kwaik and Bumann 2013). After invading the host, not only pathogens are exposed to a complex and dynamic host nutritional microenvironment with the increased inflammation and local hypoxia, but also have to deal with various metabolic dilemmas imposed by host cells to prevent nutrients from being theft. Meanwhile, in response to the ever-changing nature of the host microenvironment and successfully proliferating with the nutrients available to the host, pathogens have intelligently evolved multiple metabolic adaptations and, on the basis of these, triggered metabolic changes inside the host through a variety of common pathogenic components and specific virulence factors, which have been coordinated by a number of publications (Eisenreich et al. 2013). The reshaped metabolism involves protein, glucose, amino acid metabolism, etc. (Li and Zhang 2016), which is the embodiment of host metabolic reprogramming upon bacterial infection. In addition, the proteinogenic amino acids metabolism reprogramming of the host during bacterial infection is the spotlight of this discussion.

Amino acid is the fundamental unit of life-sustaining that contributes equally to both host and bacteria. As we all know, protein is the product of amino acid polymerization and folding modification that mediates various cellular functions. In the host, they play a central role in coordinating diverse metabolic pathways, including ATP production, nucleotide synthesis, and redox balance to achieve cellular homeostasis (Kelly and Pearce 2020). As for the bacteria, protein synthesis has been tightly linked to virulence production, pathogen growth, proliferation and immune escape (Frank et al. 2021; Hernández and Cava 2016; Radkov and Moe 2014; Vollmer et al. 2008). In summary, the study of proteinogenic amino acids metabolism reprogramming and related mechanisms of hosts during bacterial infection has guiding significance for studying the development of abnormal metabolism of hosts and treating infectious diseases, especially caused by bacterial infections.

### Glutamic acid and glutamine

Glutamine is the most abundant amino acid in plasma (Darmaun et al. 1986), which is involved in a range of biological reactions, including energy production, macromolecule synthesis, and signal transduction (Tapiero et al. 2002a). Substantial metabolism reprogramming of host glutamine can caused by bacterial colonization at the levels of individual cells, organs, and even the whole organism.

After invading macrophages, several intracellular microorganisms, including *S. aureus* (Sendi and Proctor 2008; Garzoni and Kelley 2009; Lowy 2000; Fraunholz 2012), *Brucella* (Roop et al. 2021), and *Legionella pneumocephalus* (Rathore et al. 2012), can lead to the decline of intracellular glutamine through different mechanisms. The first two can reshape the host amino acids and carbohydrate metabolism, thus, to support their own growth and survival in the host niche. l-Glutamine is the largest energy source aside from glucose. Natalia et al. ever reported decreased glucose and a parallel increase in glutamate levels after methicillin-resistant *Staphylococcus aureus* (MRSA) invasion (Bravo-Santano, et al. 2018). The metabolism reprogrammed by MRSA infection in HeLa cells was mediated by the co-activation of glutamine uptake and hydrolysis, whereas the precise mechanisms have not been explored in depth. The alterations of intracellular amino acids may be due to the involvement of amino acid metabolism or transport, or both. However, co-alterations in central carbon metabolism within cells after bacterial infection are more likely to be the consequence of lacking carbon donor precursors. As revealed by Anh et al. (Nguyen, et al. 2020), the *Vibrio parahaemolyticus* T3SS1 effector VopQ induced an reduction of non-essential amino acids, resulting from disrupted metabolism. Interestingly, to enhance growth and reproduction within the legionella-containing vacuole (LCV), VopQ can induce an up-regulated expression of SLC1A5, which is a transporter of serine, l-Glutamine, and cysteine. Similarly, Moeez et al. (Rathore et al. 2012) reported that *L.* *pneumophila* could activate NF-κB signaling in a Dot/ICM-dependent manner through LNAB inhibiting miR-23a expression, increasing glutamine level and ultimately increasing the breakdown of glutamine (Rathore et al. 2012). Changes in the cellular amino acid metabolism may profoundly affect the outcome of pathogen infection. For example, monocytes expressing surface markers of immature bone-marrow-derived suppressor cells (m-MDSCs) can be specifically aroused by *Klebsiella pneumonia* sequence type 258 (*KP ST258*) to promote a favorable immunosuppressive environment for *KP ST258*, including a significant up-regulation of l-Glutamine hydrolysis, thus, to create a favorable niche for their own survival (Wong et al. 2022). It is pointed out that even the same pathogen could cause inconsistent alterations with regard to the amino acid levels in different organs, a conclusion that is particularly applicable to *Helicobacter pylori*. The l-Glutamine in the antrum and corpus of stomach were highly depleted in gastric organoid model, but such reduction was only observed in *H. pylori*-infected mice corpus (Torres et al. 2016).

Pathogen-reshaped amino acid level is also affected by gender. The serum l-Glutamine level was decreased in *Enterotoxin Escherichia coli* (*ETEC*) infected-piglets (Ren et al. 2016), whereas the reduction magnitude was closely dependent on the sex of experimental animal. Jie et al. also showed that the l-Glutamine level in *V.* *parahaemolyticus*-colonized female abalone hepatopancreas was significantly higher than that in male’s, which may be related to the immune activation of hepatopancreas (Lu et al. 2017). This discrepancy was also detected in human. Zhihui et al. analyzed clinical samples and showed that patients with *K. pneumoniae*-associated refractory liver abscesses had reduced l-Glutamine levels as compared with patients with drainage sensitivity. So far, the mechanism by which pathogen infection caused glutamine metabolism reprogramming in animal still largely remains obscure, while we can find evidence in other species. The decline in Gln abundance caused by *Pseudomonas aeruginosa* infection with Caenorhabditis elegans is achieved by direct targeting of amino acid genes (Mahmud et al. 2020).

As the metabolite of glutamine, the level of glutamic acid and its precursor may not always vary in an opposite way, but can decrease simultaneously upon bacterial infection (Lin et al. 2020), which has a mechanism independent of the former. Eight hours after internalization of *S. aureus* in Bovine Mammary Epithelial Cells (BMEC), the abundance of glutamate was decreased, which is mediated through the inhibition of mTORC1 and the subsequent NF-κb and STAT5 pathways, leading to the inactivation of SLC1A3 (a classical transporter of glutamate and l-aspartic acid), thus, impairing BMECs' uptake of amino acids from the media (Chen et al. 2021).

### Aspartic acid and asparagine

Aspartic acid and asparagine are two non-essential amino acids with similar structures and are indispensable substrates for nucleotide synthesis (Krall et al. 2021). Both are inextricably linked to central carbon metabolism, as both glycolysis and TCA cycle provide the carbon skeleton for the biosynthesis of non-essential amino acids (Apicella and Silk 2019). Recent publication have suggested that aspartic acid and asparagine are both critical for amino acid homeostasis, maintenance of mTORC1 activity, and cellular inflammatory response (Krall et al. 2016; Knott et al. 2018; Hettmer et al. 2015). However, it should be noted that aspartic acid and asparagine do not have a single linear relationship with mTORC1 and inflammation. After bacterial infection of the host, it appears that the intracellular levels of these two amino acids and mTORC1 activity are contacted, influenced and cause-and-effect each other (Torres et al. 2016; Chen et al. 2021; Mossmann et al. 2018). In organisms, the levels of amino acids are regulated by a variety of mechanisms. Bacterial infection-reprogrammed non-essential amino acids metabolism such as aspartic acid and asparagine seems to be more related to amino acid metabolism and transport, and this discrepancy is mostly due to the bacterial virulence-related factors. For example, in *V.* *parahaemolyticus*-infected human adenocarcinoma epithelial cells (Caco-2 cells) (Nguyen et al. 2020), the biosynthesis of non-essential amino acids depleted by the bacterial T3SS1 effector VOPQ provides an indispensable carbon skeleton for the absence of metabolic disorders, such as l-aspartic acid and l-asparagine, which act in concert with the disturbance of the central carbon cycle, mAPK/ERK is activated to trigger the assembly of the NLRC3 inflammasome.

However, unlike glutamine, changes in aspartate metabolism upon bacterial infection do not appear to be dependent on sex and organ (Lu et al. 2017). Enhanced autophagy response in T cells versus epithelial cells after *S. enterica* infection were both characterized by mTORC1 inhibition, which is the outcome of exogenous asparagine deprivation via l-Asnase II (Torres et al. 2016). Likewise, the reduced l-asparagine concentration in pig serum was also responsible for the inhibition of mTORC1 activity in response to *ETEC* infection (Ren et al. 2016), but it is noted that, this inactivation in mTORC1 activity has not been clearly demonstrated to be limited to the changed abundance of l-aspartic acid and l-asparagine, and may be a consequence of metabolomic perturbation of multiple amino acids. However, for another group of bacteria, the altered mTOR activity seem to be responsible for reprogrammed amino acid metabolism. *S. aureus* is one of the main pathogens for bovine mastitis. Bacterial internalization inhibited mTORC1 activity caused a diminished expression of SLC1A3, an l-aspartic acid transporter of BMEC, leading to the reduced milk protein production (Chen et al. 2021).

### Branched-chain amino acids

As one of the essential amino acids in human body, branched-chain amino acids (BCAA) are of great physiological importance. BCAAs, including leucine, l-Isoleucine, and valine, are closely related to muscle formation, insulin regulation, amino acid synthesis and decomposition, and immune function (Neinast et al. 2019; Calder 2006). For microorganisms, it is also a vital energy source, a nitrogen donor, a feedstock for amino acid synthesis, and a key to the host defense. Studies have shown that the proliferation and virulence of several bacteria are restricted with the downregulation of BCAAs levels, indicating that these amino acids are critical for bacterial reproduction and virulence (Kim et al. 2017; Kaiser and Heinrichs 2018; Lobel et al. 2012). However, in most pathogens, the amount of BCAAs synthesized by themselves is not sufficient, let al.one the lack of ability to produce them by some bacteria. Instead, it needs to be exploited from the host (Dutta et al. 2022). Therefore, bacteria have to develop ways to obtain enough BCAAs for their growth. That is why BCAA levels in the host, in most cases, decrease in the short-term infection. Although bacteria-triggered BCAAs reduction has been reported, the underlying mechanisms about how these microbes affect BCAAs and the host physiology still remains to be explored.

One of the strategies for bacteria to acquire BCAAs is through a bunch of transporters to directly absorb them from the host (Saier 2000). Julienne et al. have shown that *S. aureus* can obtain BCAAs from the host via the transporters BRNQ1 and BRNQ2, where the former has a high affinity for all BCAAs, and is the main transporter during *S. aureus* infection. In contrast, while BRNQ2 can only specifically deliver l-Isoleucine (Kaiser et al. 2015). Other microorganisms can also utilize BRNQ transporters for BCAAs transportation, including *Bacillus subtilis*, *Lactobacillus*, and *Corynebacterium glutamicum* (Stucky et al. 1995; Belitsky 2015; Tauch et al. 1998). BRNQ, BCAP and BRAB are the major BCAAs transporters in *Bacillus subtilis*. It is noted that BCAAs is critical for the virulence of *S. aureus USA300* and *B. subtilis*, this regulation of bacterial virulence may be related to the intracellular level of Cody effector molecules (Kaiser et al. 2015; Belitsky 2015). Apart from the above mentioned transporters, SLC7A5 has also been identified to serve as a leucine transporter in BMEC, while it can be inhibited by *S. aureus* to increase the free leucine in serum (Chen et al. 2021). Other pathogenic bacterium such as *Francisella* can extracellularly acquire the l-Isoleucine through the protein FTN-1654, a *Francisella*-specific l-Isoleucine transporter (Gesbert et al. 2015). In the *Streptococcus pneumoniae*, an ABC transporter called LivJHMGF has also been identified to be responsible for BCAAs transportation, and is necessary for the *S. pneumoniae* to cause disease (Basavanna et al. 2009).

Another common mechanism for bacteria to obtain BCAAs is through the activation of autophagy response in the host, thus, to transiently increase the abundance of extracellular free amino acids. Many pathways can activate autophagy, among which *Salmonella* and *Shigella* triggered the amino acid starvation of the host by causing membrane damage, which subsequently inhibits mTOR signaling, and ultimately leading to the activation of autophagy (Tattoli et al. 2012). Notedly, in addition to being a substance produced by autophagy, leucine itself can also serve as a switch to initiate the mTOR pathway (Wolfson and Sabatini 2017; Nicklin et al. 2009).

Bacteria-upregulated BCAAs abundance has been also reported using clinical samples. Children diagnosed with pneumococcal pneumonia due to *S. pneumoniae* infections have elevated levels of BCAAs in their pleural fluid (Chiu et al. 2016). An increase in BCAAs was also observed in the gill cells of female abalone after infected with *V.* *parahaemolyticus* (Lu et al. 2017). The increased BCAAs maybe related to the host immunity as these amino acids are vital for maintaining immune homeostasis, and the immune protein synthesis (Calder 2006).

In addition to these pathways, bacteria can affect BCAAs metabolism in several other approaches. For example, human macrophage-like cells infected with *Brucella abortus* undergo a perturbed metabolism with the attenuated TCA cycles and the reduced amino acid consumption, resulting in amino acids accumulation in a short period. Meanwhile, the expression of enzymes related to amino acid metabolism increased in infected cells, thus competitively robbing the host amino acids (Czyz et al. 2017). In contrast, *L.* *pneumophila* exploits the conserved eukaryotic process of multiubiquitylation of K48 junctions and the proteasomal machinery through the AnkB true F-box effector to generate amino acids (Gesbert et al. 2015). In addition, *H. pylori*-infected gastric cancer cells (MKN-28) showed an increased level of BCAAs, which is correlated with the type 2 diabetes, obesity and Alzheimer's disease, but the underlying mechanism is largely unknown (Cuomo et al. 2020).

### Arginine

l-Arginine is a semi-essential amino acid, an intermediate of urea cycle, and it also needs to be ingested from the diet. Arginine is critical for the host to defense against pathogen, as evidenced by the fact that such amino acid is a key substrate for NO biosynthesis (Tapiero et al. 2002b), and it can be exploited by macrophages to kill invasive microorganisms (Mills 2012), and the availability of arginine in the external microenvironment is important for T cell activation (Geiger et al. 2016).

Successful establishment of infection is always achieved by suppressing the host innate immune response and enhancing the pathogen virulence, both of which are tightly related to the metabolism of arginine. Upon *M. tuberculosis* infection, ARG2, mainly distributed in the dendritic cell, can be highly induced by microRNA-155 inhibition, therefore, to control the arginine concentration in the extracellular environment, and eventually affecting T cell activation and immune evasion (Shi et al. 2019). Such process was also inversely validated in a mouse model of *H. pylori*-induced gastritis (Keilberg et al. 2021) and reduced arginine levels was also detected in the serum (Lin et al. 2020).

For bacteria which cannot synthesize arginine, it is indispensable for them to exploit the amino acid from the host to support their growth and virulence. Piet et al. established a *S. pneumoniae* strain deficient in arginine biosynthesis. As compared with wild type bacteria which could convert the host's citrulline to arginine, the pathogen titers in both cerebrospinal fluid and blood after 6 h infection is down-regulated as well as the patient prognosis and arginine concentration in serum. Similarly, the full-length *speA* gene encoding arginine decarboxylase is essential for the virulence of *Franciscan A. I* Strain, which is considered to be the most virulent bacterial pathogen known. It is worth noting that arginine is a well-recognized activator of the mTOR pathway (Lu et al. (2017), Mahmud et al. (2020); Oshiro et al. 2014; Meng et al. 2020) however, the pathophysiological influence on the host due to bacterial infection is still lacking.

### Threonine

Threonine as an essential amino acid in mammal (Meltzer and Sprinson 1952) is important in maintaining intestinal mucosal integrity and function (Law et al.. 2007), promoting protein synthesis, regulating lipid metabolism (Kramsch et al.. 1971; Kamata et al.. 1995), and enhancing immune homeostasis (Mao et al.. 2011). The abundance of threonine tend to be decreased upon bacterial infection. For example, perturbation in central carbon metabolism by *V.* *parahaemolyticus* infection is one of the vital approaches to enhance its infection effect, leading to the declined production of amino acids from glucose as well as the two essential amino acid tryptophan and threonine. The same applies to the changed amino acid abundance in human macrophage-like cells (THP1) infected with *Brucella* (Czyz et al.. 2017) rand Hela cells challenged with *S. aureus* (Bravo-Santano et al.. 2018). Predictably, disrupting the host metabolism is not the goal for colonized bacterium. Rather, exploiting the host for nutrients to achieve a survival advantage is the main reason, which can be explained by the prevalent reduction of essential amino acids. In accordance, a significant depletion in the serum abundance of 14 amino acids such as threonine caused *H. pylori* infection has been reported, most of which are prominent in the maintenance of host immune function. (Lin et al.. 2020). Such results were replicated in the mice model revealed by Daniela Keilberg et al.. (Keilberg et al.. 2021). However, the decreased abundance of threonine was not applied to all kinds of bacterium. A subset of microbes, such as *L.* *pneumophila* (Rathore et al.. 2012; Eisenreich and Heuner 2016) and *bacilli septicemia* (Xiao et al.. 2020), tends to significantly elevate certain amino acid concentrations, the former includes threonine and l-Cysteine, while the latter consists of glycine and serine.

### Tryptophan

Tryptophan, an essential amino acid, is capable of activating the aryl hydrocarbon receptor (Ahr) through binding with its metabolite kynurenine, leading to the activation, proliferation, and differentiation of T cell, and resultingly contributing to the maintenance of normal immune function (Platten et al.. 2012; Grohmann and Puccetti 2015; Mondanelli et al.. 2019). The availability of tryptophan in the external nutritional microenvironment is critical for the successful bacterial infection of the host (Munn et al.. 1999; Ren 2018), as evidenced by the declined tryptophan level in the serum of infected mice (Lin et al.. 2020). Therefore, it is suggested that the down-regulation of tryptophan metabolism and transportation may be beneficial for the pathogenesis of bacterial infection, which has been evidenced by *H. pylori* (Keilberg et al.. 2021), *L.* *pneumophila* (Eisenreich and Heuner 2016), and *P. aeruginosa* (Mahmud et al.. 2020), respectively. The metabolic reprogramming of tryptophan, involving the alteration of amino acids and tricarboxylic acid cycle metabolism in the host, has been further investigated in *V.* *parahaemolyticus* (Nguyen et al.. 2020) and *B. abortus* (Czyz et al.. 2017) infected models. Distinct from the other bacteria, which obtain a comfortable living environment by directly or indirectly reshaping the host metabolism, *L.* *pneumophila* has evolved the ability to modify its morphology and metabolism to adapt to the dynamic nutritional condition (Fonseca and Swanson 2014; Al-Quadan et al.. 2012), which may represent a novel strategy for microbes to achieve intracellular colonization.

Research focusing on the reprogramming of host tryptophan metabolism caused by bacterial infections has not only been limited to cell experiments, but also extended to the clinical studies. By including children with Complicated parapneumonic effusions (CPE) and non-CPE caused by *S. pneumoniae* infection, the investigators revealed elevated tryptophan levels in the pleural fluid samples from CPE patients (Chiu et al.. 2016), whereas the underlying mechanism has not been fully explored.

### Lysine

Lysine is also a type of essential amino acid for mammals. Positive nutritional effects of lysine include improving fat oxidation, boosting the body's immune system (Kornegay et al.. 1993), and defensing against viral. To survive and proliferate within the host cells, most facultative and obligate intracellular pathogens choose to compete with the host for resources, such as central metabolites and amino acids. Both *Brucella* and *Salmonella* are intracellular reproducing microorganisms, and research has revealed that cells infected with these two pathogens prefer to consume less amino acids while accumulating more nutrients intracellularly, due to the reshaped pattern for utilizing amino acids and glucose by microorganisms (Czyz et al.. 2017; Jiang et al.. 2021). Likewise, an increase in intracellular lysine abundance in *V.* *parahaemolyticus*-infected host cells was reported, which may be correlated with the osmoregulation (Nguyen et al.. 2020). It has now been established that lysine and its byproduct through decarboxylation, cadaverine, can both mitigate oxidative stress. LdcF-deficient *Francisella tularensis* extends its survival through inhibiting lysine decarboxylation, promoting lysine accumulation and cadaverine reduction, and thereby enabling the host cell to defense against stress oxidation (Felix et al.. 2021; Tomar et al.. 2013).

### Serine

Serine is a non-essential amino acid that plays a crucial role in the metabolism of fat and fatty acids. The abundance of multiple amino acids, including serine, usually declined significantly upon pathogen infection. For instance, *S. septicum*-challenged cells showed an altered protein spectrum, among which proteins expressing enzymes related to serine metabolism were significantly upregulated, resulting in an increase in the activity of these enzymes and the serine metabolism rate (Xiao et al.. 2020). Similarly, a variety of amino acids such as serine and glycine were reduced in *H. pylori*-infected mice (Keilberg et al.. 2021). In most situations, this reduction was due to the changed amino acid transportation. *L. pneumophila*-specific type 4B secretion system (t4bSS) is required for the establishment of replicative legionella-containing vesicles (LCV), and after infection of human monocytes, *L. pneumophila* induces the expression of the amino acid transporter protein SLC1A5, which is transported to LCV and facilitates the entry of neutral amino acids, such as serine and cysteine into LCV (Eisenreich and Heuner 2016). A similar situation exists during infection by *Salmonella typhimurium*, where the effect on serine synthesis is mediated by the bacterial protein SopE2 (a type III secretion system effector encoded in the pathogenicity island SPI-1). Following infection of macrophages by *S. typhimurium*, metabolomic data suggested an enhanced glycolysis and reduced serine synthesis in the infected macrophages (Jiang et al.. 2021).

### Glycine

Glycine is a key non-essential amino acid for both leukocyte proliferation and antioxidant defense (Li et al.. 2007). Infection of rabbit bronchial cells by *B. septicum* caused the activation of the glycine pathway, leading to an increase in intracellular glycine level (Xiao et al.. 2020). However, such elevation was not seen in every microorganism infected cells. For example, a marked reduction of glycine was reported in *H. pylori* and *Vibrio parahaemolyticus* infected cells (Nguyen et al.. 2020). *V.* *parahaemolyticus*-secreted T3SS1 effector VopQ extensively reshaped the host’s metabolism of amino acids and carbon, and caused significant metabolic abnormalities in infected cells. This occurs frequently in the nature world. *ETEC* infection lowered the concentration of isoleucine and non-essential amino acids, such as glutamine, asparagine, citrulline, and ornithine in piglet serum, while increased the levels of glycine and aminobutyric acid. The underlying mechanism of this effector has been described in two ways: one suggests that it changes the rate of amino acid transport, while the other revealed a declined synthesis of non-essential amino acids due to the lack of carbon donor precursors. In addition to influencing amino acid metabolism, the effector VopQ can modify the host immune system by stimulating autophagy in both phagocytic and non-phagocytic cells (Higa et al.. 2013; Burdette et al.. 2009).

### Histidine

Histidine, an alkaline amino acid, is mostly synthesized through decarboxylase by histidine deaminase, and considered to be an essential amino acid in human. This amino acid can be utilized as a nutrient for supporting bacterial survival, as it has been revealed by Daniela et al.. that histidine level in organoids derived from the stomach antrum and corpus was decreased after *H. pylori* infection (Keilberg, et al.. 2021). The reduction of histidine may also be triggered by bacteria-related metabolic reprogram, leading to the modified host metabolism and the activation of autophagy response. Take *S. aureus* as an example, l-Phenylalanine can be synthesized by *MRSA* from 3-phenylpyruvic acid and histidine through histidine-phosphate transaminase (Bravo-Santano et al.. 2018). The host cell's histidine is consumed not just as an energy source for the bacteria, but also as a donor for l-Phenylalanine. Likewise, *B. abortus*-mediated perturbation of host mitochondrial function prohibits the entry of any amino acid, including histidine, into the TCA cycle (Czyz et al.. 2017). Histidine in the gut microbiota also produces a variety of metabolite molecules that affect the host, such as imidazole propionic acid (IMP), which is elevated in T2MD and impairs insulin signaling by activating the p38 γ-p62–mTORC1 pathway.

### l-Cysteine

l-Cysteine is a common sulfur-containing amino acid in mammals. The most important function of l-Cysteine is to scavenge free radicals, antioxidants, and copper integrators. Microorganisms can gain energy from the carbon chain of l-Cysteine through desulphurases, such as methionine that can be converted into α-keto-butyrate, ammonia and methanethiol. The underlying mechanism of bacteria-imposed host cell damage may be due to the rapid ingestion of large amounts of thiocysteine CySS, then converting them into cysteine Cys. Meanwhile, free Fe^2+^ drives the conversion of Cys to H_2_O_2_ and catalyzes the formation of OH. Fenton reaction driven by endogenous cysteine in *E. coli* may promote drug resistance. Moreover, to improve legionella growth and reproduction, SLC1A5 (neutral amino acid specific), an amino acid transporter, was highly induced to express on LCV membrane, and promote the absorption and transport of serine, l-Glutamine or cysteine (Eisenreich and Heuner 2016).

### Methionine

Methionine is a sulfur-containing essential amino acid, which is closely related to the metabolism of various sulfur-containing compounds in the organism. Methionine is the most abundant sulfur-containing amino acid, and is the main supplier of sulfur for the host. Methionine is able to convert into l-Cysteine and glutathione, which acts as an antioxidant and antidote in the body, helping to ward off cell damage caused by the highly oxidative environment created by bacteria. Pathogen infection usually caused an accumulation of methionine in living cells, which has cytoprotective and antioxidant functions (Bender et al.. 2008). For example, *V.* *parahaemolyticus*-infected male and female abalone both showed elevated methionine levels (Lu et al.. 2017). Methionine is essential for maintaining the normal level of folate in host and bacteria. For example, metformin-induced damage to the methionine cycle in *E. coli* leads to the increased synthesis of SAMe, thus, restricting methionine production by blocking methylene THF reductase, inhibiting the *E. coli* folate cycle and extending host life (Induri et al.. 2022).

### Proline

Proline as an non-essential amino acid is a fundamental substrate for almost all proteins. The main function of this amino acid is to help the body to decompose proteins. Meanwhile, it is an critical component of collagen.

Proline is involved in metabolism as a nutrient. Microorganisms such as *E. coli,* Gram-negative *S. enterica*, and *Klebsiella spp*. can utilize it as a sole nitrogen or carbon source, leading to a decrease in the host proline level (Moses et al.. 2012; Chen and Maloy 1991**).** Moreover, as revealed by Faiza et al.., this amino acid could also serve as a osmoprotective agent (Zaprasis et al.. 2013). Proline is also indispensable for bacteria to defense against temperature and oxidation. Lagautriere et al.. identified the proline metabolic pathway is necessary for the persistence of *M. tuberculosis* in the host (Lagautriere et al.. 2014). Pathogen-related metabolic reprogram usually causes a decrease in proline level. For example, microorganism such as *B. abortus* infection results in the disruption of host mitochondrial function, thereby affecting the host amino acid metabolism. The infected cells are unable to metabolize any amino acids entering the TCA cycle, but interestingly, host glucose metabolism is not affected (Czyz et al.. 2017). Reduction of total amino acid content due to the decreased host amino acid transport during *P. aeruginosa* infection, including proline, may be mediated by the addition of γ-glutamyl groups to γ-glutamyltransferases (Mahmud et al.. 2020).

### Phenylalanine/tyrosine

As an aromatic amino acid in human body, most of l-Phenylalanine is oxidized to tyrosine by PAH catalysis, producing l-Phenylalanine or tyrosine-derived dopamine in the gut, which plays an important role in controlling movement and mood. A significant increase in the ratio of l-Phenylalanine to tyrosine could be an indicator of the inflammatory activity.

*MRSA* can synthesize l-Phenylalanine from 3-phenylpyruvic acid and histidine, resulting in differences in host histidine and l-Phenylalanine levels. In *Klebsiella pneumoniae*-infected hosts, enrichment pathway analysis revealed the activation of host metabolism of arginine, l-aspartic acid, and l-Phenylalanine. Human macrophage-like cells infected with *B. abortus* showed decreased TCA cycle metabolism and reduced amino acid consumption as well as the hindered capability of host cells to utilize amino acids. Likewise, an increased abundance of phenylalanine, leucine, l-Isoleucine and threonine was detected due to *S. pneumoniae* infection, whereas the underlying mechanism still remains unknown.

l-Phenylalanine and tyrosine could also be used as energy sources by pathogens. The levels of l-Phenylalanine, tryptophan and tyrosine in the liver and pancreas of female abalone infected with *Vibrio* decreased significantly. Similar phenomenon also observed in *H. pylori*-infected gastric corpus organoids.

## Conclusions and discussion

Within our review, we undertake a comprehensive examination of alterations occurring in the host's amino acid profile subsequent to pathogenic invasion—an area of substantial significance within the domain of pathogen–host interplay investigation. Through meticulous scrutiny of an array of illustrative case studies, we endeavor not only to unveil the intricate panorama of these modifications, but also to glean profound insights into their pivotal involvement in orchestrating the immune retort and intricacies of infection-associated metabolism.

**Fig. 1:**
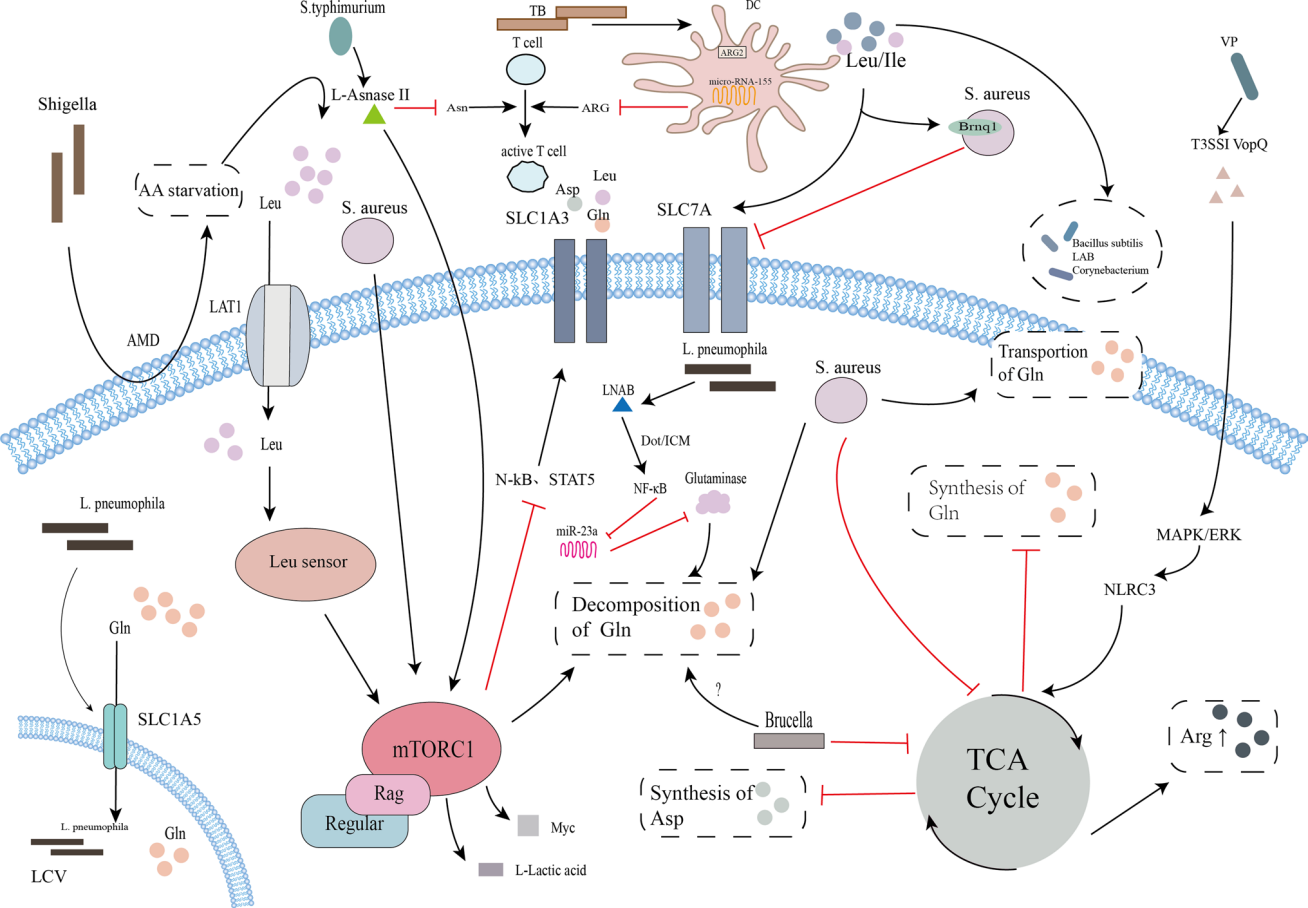
AA starvation: amino acid starvation; AMD: membrane damage; ARG: arginine hydrolase; Arg: arginine; Asn: asparagine; Asp: asparagine; DC: dendritic cells; Gln: glutamine; LCV: Legionella-containing vesicles; Leu: leucine; *L. pneumophila*: *Legionella pneumophila*; MRSA: methicillin-resistant *Staphylococcus aureus*; *S. aureus*: *Staphylococcus aureus*; *S. typhimurium*: Salmonella typhimurium; SLC: solute carrier; TB: *Mycobacterium tuberculosis*; TCA Cycle: tricarboxylic acid cycle; Ile: isoleucine; VP: *Vibrio parahaemolyticus*

Evident from our meticulous review is the unequivocal capacity of bacterial incursion to intricately perturb the host's amino acid metabolism, orchestrating this modulation through a panoply of mechanisms—ranging from amino acid uptake, synthesis, transport, utilization, to hydrolysis. This overarching influence extends far and wide, encapsulating the precise targeting of genes encoding pertinent amino acids, the recalibration of central carbon metabolism, the dynamic activation or restraint of autophagic processes, and the nuanced modulation of amino acid synthesis, transporters, enzymatic kinetics, and sensory apparatuses. The landscape thus unfurled was marked by discernible differentiations in the patterns of amino acid alterations, exhibiting distinct signatures tailored to diverse pathogenic agents and host entities. These cascading perturbations find their resonance in the intricate web of host cell metabolic pathways, profoundly steering their energy provisioning dynamics and immune responsiveness (Fig. 1). This symphony of alterations holds within its grasp the potential to precipitate a plethora of pivotal outcomes within the realm of infection, encapsulating a spectrum of arenas including but not limited to the following five aspects.

The first aspect centers on the orchestration of the immune response: nuanced shifts in specific amino acids hold the potential to intricately modulate the regulatory framework governing the host's immune machinery. These select alterations within amino acid profiles have the potential to serve as catalysts, potentially setting in motion a cascade of events culminating in the activation of immune cells and consequent amplification of the inflammatory cascade. This intricate phenomenon is intricately orchestrated by manifold mechanisms, encompassing the potentiation of immune cell activation, the induction of cytokine release, and the judicious orchestration of T cell responsiveness. Significantly, l-Glutamine , heralded for its pivotal role as an energetic substrate within immune cells, emerges as a pivotal factor dictating cellular proliferation kinetics and activation status. The delicate perturbations in l-Glutamine provisioning unveil its potential to profoundly influence immune cell functionality and the finely tuned equilibrium of immune responsiveness (Newsholme et al.. 1999). Similarly noteworthy, Arginine, positioned as a linchpin within this narrative, assumes multifaceted roles. Certain pathogens adroitly leverage Arginine and the machinery of arginine synthetase to circumvent the host cell's immune response and signaling machinery (Bronte and Zanovello 2005). Cysteine, a sulfur-containing amino acid, participates in the synthesis of glutathione within cells, thereby influencing the cellular redox state. Variations in cysteine supply can potentially impact the antioxidant capacity and immune activity of immune cells (Droge and Breitkreutz 2000).

The subsequent dimension entails host metabolic reconfiguration: select amino acid shifts wield the potential to intricately navigate the labyrinthine network of host metabolic pathways. This engenders a potential for the orchestrated rerouting of metabolic trajectories, instating an environment more conducive to pathogenic proliferation. Concurrently, these metabolic recalibrations may impose a transformative influence upon the host cell's energy provisioning and nutrient requisites—strategically accommodating pathogen survival and replication. To illustrate, l-Glutamine , a cardinal participant in normative metabolic orchestration, emerges as a prominent protagonist within the realm of infectious diseases. The escalated requisites inherent to immune cells and other swiftly multiplying entities drive an amplified demand for l-Glutamine , a requirement to satiate their escalated energy and nitrogen essentials. This dynamic engenders augmented l-Glutamine consumption, instigating a domino effect of metabolic modifications within the host milieu, calibrated to satiate the burgeoning exigencies of the immune response (Curi et al.. 2005). Arginine, an intrinsic player in this narrative, finds itself enmeshed within the realms of infection. Here, potential disruptions in Arginine metabolism reverberate into the precincts of the host's disease resistance. Within the contours of inflammatory milieus, Arginine's roles diversify to encompass the generation of reactive nitrogen species, thereby precipitating a scenario, wherein insufficient Arginine provisioning culminates in compromised immune cell activity (Wu and Morris 1998). Similarly, Tryptophan, a cornerstone in serotonin and melatonin production, succumbs to perturbations within its metabolic stride during infection. For instance, the action of Nitric oxide synthase can potentially engineer a recalibration within the Tryptophan metabolic pathway, leading to a depleted Tryptophan supply. This intricate cascade has the potential to exert cascading repercussions upon host immune regulation and cellular signaling (Munn and Mellor 2007). In parallel, the saga unfolds for l-Cysteine, which might be coerced into a heightened requisition to combat oxidative stress. This cascading demand may inadvertently trigger a diminution within l-Cysteine provisioning, ultimately underscoring its potential to modulate glutathione synthesis and, concomitantly, the delicate equilibrium encapsulated within the cellular redox landscape (Droge 2002). Finally, within the complex interplay of inflammation and infection, Methionine's metabolic trajectory is not immune to transformation. These shifts can transmute the trajectory of Methionine metabolism, ushering perturbations within its very fabric and inducing nuanced shifts in the production of select inflammatory mediators (Jeckel et al.. 2014).

Third, a pivotal aspect emerges in the form of the modulation of bacteria–host interactions, wherein host amino acid variations can profoundly influence the intricate interplay between pathogens and their host environment. This encompasses a gamut of processes spanning attachment, invasion, and circumvention of host defense mechanisms orchestrated by pathogens. The ramifications are far-reaching: select amino acid modifications potentially engender transformations in the molecular landscape of host cell surfaces, thereby conferring potent influence upon the interplay with pathogens. Arginine assumes a paramount role in this discourse—certain pathogens adeptly exploit arginine and the machinery of arginine synthetase to intricately subvert the host cell's immune retort and signaling conduits (Hung et al.. 2011). Similarly, l-asparagine, by virtue of its strategic positioning, holds sway over the adhesive and invasive comportment of bacteria within the milieu of bacteria–host interplay. Some pathogens astutely harness l-asparagine within host cells as a host-driven strategy for propagation (Shah and Swiatlo 2008). Equally discernible, methionine surfaces as a modulator of import—some pathogens deftly utilize the host cell's methionine metabolism pathway to obfuscate the functional and signaling modalities of host cells, thus orchestrating a nuanced manipulation of the pathogen–host interplay (Kipkorir et al.. 2021).

Fourth, the interplay of inflammation and disease progression assumes prominence, as distinct amino acid perturbations may catalyze the escalation of immune-driven inflammation, thereby augmenting the vulnerability to tissue damage. The protraction of this inflammatory milieu further precipitates the trajectory towards disease emergence, encompassing entities, such as chronic inflammation-related disorders. Arginine, its metabolites, such as nitrite and polyamines, emerge as pivotal players in immune inflammation orchestration. Notably, certain investigations posit that undue arginine intake may potentiate immune inflammation, especially under specific chronic inflammatory contexts (Wu and Morris 1998; Morris 2010). Similarly, Methionine, a sulfur-enriched amino acid, forges integral links within diverse biosynthetic cascades within immune cells. In-depth examinations have illuminated a correlation between Methionine metabolism and the modulation of inflammatory response and immune cell activation, potentially influencing immune inflammation dynamics (McGaha et al.. 2012). Lysine, an indispensable amino acid, entwines within multifarious cellular processes, including protein modifications and immune retorts. An equilibrium disruption in lysine and other amino acid provisioning potentially assumes a role within select inflammatory ailments (Smriga et al.. 2002).

Fifth, a pivotal dimension emerges in the form of the influence exerted upon drugs and therapeutic strategies: host amino acid fluctuations bear the potential to recalibrate drug metabolism and mechanisms of action, consequently reverberating through the spectrum of infection management. Variations within host amino acids can wield a potent influence over drug absorption, distribution, metabolism, and excretion, underscoring the necessity of a comprehensive comprehension of these dynamics to optimize drug selection and therapeutic regimens. The spotlight falls on Lysine, whose metabolism and concentration might influence drug–protein binding. As certain pharmaceutical agents operate through protein binding, alterations in host lysine levels can potentially modulate drug–protein interactions (Hacker et al.. 2017). In addition, leucine, a crucial branched-chain amino acid, might intricately participate in drug absorption and distribution, thereby potentially influencing the bioavailability and tissue dispersion dynamics (Emami et al.. 2012). Within this gamut, l-Glutamine surfaces as an Essential amino acid, pivotal within diverse host biological processes. Interactions between drug and l-Glutamine metabolism within the host milieu may potentially intertwine, ultimately influencing drug metabolism and therapeutic efficacy (Slominski et al.. 2002).

In summation, amino acids wield a pivotal role within the intricate mosaic of bacterial infection dynamics, encompassing realms of immune modulation, metabolic sustenance, protein synthesis, and an array of other biological facets. The prospect emerges that the targeted modulation of amino acid metabolism for specific infectious entities holds the potential for innovative avenues in research, promising advancements in disease mitigation, treatment, or prophylaxis. This modulation can potentially ameliorate disease symptoms, buttress immune functionality, and temper inflammation, collectively exerting a constructive impact. Amino acids such as arginine, l-Glutamine , and l-Cysteine stand out as candidates with potential roles in immune regulation and inflammation attenuation. The meticulous regulation of these key amino acids' metabolism could potentially serve as an instrumental lever to intervene in infection progression and immune inflammation. Furthermore, amino acid supplementation might serve to bolster cellular energetics and metabolic fortitude, thereby bolstering the host immune repertoire.

However, salient considerations should be kept in view. The intricacies at play in infection initiation and development typically stem from a nexus of factors, with amino acids merely comprising one facet of this complexity. Moreover, individual variability, encompassing divergent metabolic statuses and immune responses, underscores the necessity for personalized treatment approaches tailored to specific circumstances. The validation in clinical settings is imperative, as while certain investigations have offered insights into amino acids' potential role in infectious diseases, a robust body of clinical evidence is requisite to substantiate their efficacy and safety. Strategic therapeutic modalities that harness amino acids, whether as adjuncts, prophylactic strategies, or primary therapies, must be rigorously assessed in terms of their potential benefits, risks, and compatibility with conventional therapeutic paradigms. We are compelled to explore the potential adjunctive application of amino acids alongside antibiotics.

The intricate regulatory interplay of amino acids and their metabolites, within the backdrop of infection, encapsulates the transmission of signaling molecules within the infection microenvironment, the acquisition of proliferative prowess, and the elicitation of immune responses. Consequently, future explorations must be oriented towards comprehending the core enzymes of amino acid metabolism, delineating the manifold signaling cascades emanating from amino acid metabolism, and understanding their intricate intersection with glucose and lipid metabolism. This trajectory serves as a foundation for unveiling the intricate sensing mechanisms and feedback loops governing amino acid metabolism within the tapestry of infection progression, encompassing its expansive clinical applicability.

## Data Availability

Data availability is not applicable to this article as no new data were created or analyzed in this study.
